# Sense of loneliness of veterans in Southern Iran: a cross-sectional study

**DOI:** 10.1186/s12889-023-15196-8

**Published:** 2023-02-07

**Authors:** Abdolrahim Asadollahi, Mehdi Mojadam, Maria Cheraghi, Mariam Hasanshahi, Narges Nazari, Alimohammad Keshtkar, Aliasghar Arastoo, Morteza Abdulatif Khafaie

**Affiliations:** 1 Dept. of Health Promotion & Gerontology, Faculty of Health, Shiraz University of Medical Sciences, Shiraz, Iran; 2 Dept. of Public Health, Faculty of Health, Abadan University of Medical Sciences, Abadan, Iran; 3 Social Determinants of Health Research Center, Ahvaz Jundishapur University of Medical Sciences, Ahvaz, Iran; 4 Dept. of Public Health, School of Health, Ahvaz Jundishapur University of Medical Sciences, Ahvaz, Iran

**Keywords:** Social isolation, Loneliness, Veterans, Iran

## Abstract

**Aims:**

More than three decades have passed since the end of the Iraq-Iran war, and this period has been concurrent with a rapid growth of the older community in Iran which includes the community of veterans who often suffer from serious psychological and behavioral problems. The study aimed to investigate the prevalence and determinants of loneliness in middle and older veterans in southern Iran.

**Materials & methods:**

A cross-sectional study conducted among 583 middle and older male veterans (50 years <) who were selected by the census method in 2021. Data collection was done using UCLA Loneliness Scales. The collected data were entered into SPSS version-26 and Amos-24 and analyzed by multivariate ANOVA, multivariate regression, and structural equation at a threshold significance level of 0.05.

**Findings:**

The mean score of feeling lonely in the veterans was 51.08 ± 4.74. The study found a significant relationship between the participants’ education and their feeling of loneliness (P-value = 0.01, effect size = -0.31). Multivariate regression demonstrated that demographic variables (i.e., age, employment status, level of education, type of living) and the severity and type of injury predict 31% of the variance in the middle and older veterans’ feeling of loneliness.

**Conclusion:**

The mental, psychological and physical effects of war disability were associated with the social functions of veterans in the family and community. Strategies such as increasing social support and psychological counseling for veterans along with improving their pension and income can be effective in promoting public health, especially the mental health of this group.

## Introduction

One of the most important challenges of the present century is the aging of the population and the increased older adults’ population. The rise in human life expectancy and the increased older adult population are one of the achievements of the last century, rendering population aging a phenomenon that some human societies have to wrestle with [1]. The older adults contribute to 10% of the world population and it has been projected to rise to 16% (equal to 1.6 billion) by 2050, with more rise in low- and middle-income countries driven by the momentum of past growth [2, 3] For instance, the proportion of older adults in Eastern and South-Eastern Asia is around 13% in 2022 projected to almost double by 2050 [3]. In Iran, according to previous studies, about 11% of the population are older adults, and projected to rise to 30% by2050 [4].

Like many other social phenomena, the aging of the population needs to be examined concerning the possible differences in terms of the macro-social structures. The trends observed in the aging of the population at a macro level of society may be fundamentally different from its subgroups. One of the most important differences in these subgroups in terms of population aging is related to the lived experience of older adults. That is, the lived experience of older adults with physical and psychological injuries cannot be considered as a similar transition and shared experience with otherwise normal older people in society.

Now, more than three decades have passed since the end of the eight-year Iran-Iraq war in the 1980s, the longest war of the twentieth century, and the survivors and the wounded of which have now entered old age with a rather difficult experience. It is predicted that in 2021, the proportion of the older adults population of veterans will reach more than 75% of the total population of veterans, and if we assume an earlier onset of aging due to the physical and psychological impact of war and disability on the aging process among this group, the older adults population of veterans will be much larger [5].

As a psychological stressor, war results in a broad array of consequences on society in various individual and social contexts. The physical and mental problems of the veterans and their effects on the lives of these individuals are among the worst consequences of this phenomenon [6]. If not diagnosed in a timely and prompt manner and the necessary treatment measures not taken accordingly, psychological injuries like physical injuries, will have adverse, severe, and visible effects on the mental health of veterans [7]. Psychological problems are observed in varying degrees and with considerable frequency in veterans, especially in middle and older veterans. Disorders such as stress, depression, memory loss, change in sleep patterns, and feelings of loneliness are among these problems [8]. Feeling lonely is an intellectual reality which means that the individual is independent of the known values ​​in society. In such a situation, the individuals are alien to their social contexts, are isolated from the conventional values ​​and goals of society, and do not consider themselves in line with what is acceptable and important to society [9].

Studies show that 44% of American veterans aged 60 and overexpress dissatisfaction with loneliness in their lives, and 10.4% of these people state that they often feel lonely and isolated and do not want to be in public [10]. A study conducted in Fars province, south of Iran showed that more than one-third of veterans with one blind eye suffer from a variety of mental health problems. More than three-quarters of these individuals had two or more simultaneous disorders, among which anxiety and depression were more common [11]. In another study, the prevalence of depression in all veterans was reported to be 71%, and this prevalence was higher among casualties of chemical warfare compared with other veterans [12].

Lack of relationships that serve social functions with others poses a serious threat to the well-being and health of the individuals, and those who do not maintain strong and meaningful relationships with others and do not have someone to turn to when necessary, are largely immersed in themselves. This often leads to dangerous problems such as mental disorders, depression, low self-esteem, social problems, and physical symptoms [13], and dissatisfying relationships serve as a social factor. Consequently, low levels of social activity, limited social networks, poor self-perceived health, and depression assumed as important determinants of loneliness [14]. The period of the Iran-Iraq war has been concurrent with a rapid growth of the older community in Iran which includes the community of veterans who often suffer from serious psychological and behavioral problems. However, little, if any, scholarly attention has been paid to this issue. Therefore, preventing this increase in mental disorders must be a research priority that will let us make informed and rational decisions regarding the situation of middle and older veterans and their problems. Thus, the purpose of this study is to investigate the predictors of loneliness in middle and older veterans in southern Iran to help policymakers and planners in their duties.

## Materials & methods

The present study was a cross-sectional study conducted at the outset of the year 2021. The study included 583 combat veteran’s males over 50 years, living in one of the southern provinces in Iran (namely: Fars, Bushehr, Hormozgan, Kohgiluyeh & Boyer-Ahmad, and Khuzestan) who were selected by census method. The participant that was literate, not under clinical treatment, and willing to participate included in the study. Two subjects that had major depression and were under an antidepressant agent’s treatment were excluded from the study. The instrument used in this study was a standard UCLA Loneliness Scale (version 3, 1996), 20 items which consisted of two sections. The scale generally uses four response categories: never, rarely, sometimes, and often. Socio-demographic information of participants (i.e. age, employment, education, and type of living), and combat injury profiles (i.e. type and severity of injury) were obtained. The amount of injury or disability of the veteran is determined by the medical councils of the armed forces. The 20-item UCLA loneliness questionnaire (including half positively worded items e.g., “I am an outgoing person”, and half negatively worded items e.g., “I feel left out”) was used to measure loneliness. In this scale, the answers to the questions are based on a four-point Likert, with participants responding “never = 1”, “rarely = 2”, “sometimes = 3” and “always = 4”. The scores of this scale range from 20 to 80, and higher scores indicate a greater frequency of feelings of loneliness. However, scores greater than 40 are usually considered as representing actual loneliness [15]. The validity of this scale was tested parallel to Beck’s Depression Inventory, and a correlation coefficient of 0.77 was obtained [16]. Cronbach’s alpha values of 0.76 and McDonald’s Omega score of 0.71 indicate acceptable internal consistency for the UCLA Loneliness Scale in this study. After obtaining the necessary permits from the Vice Chancellor for Research of medical universities in the correspondent regions where the study was conducted.

The researchers referred to the participants in person and after introducing themselves and stating the purpose of the research and reassuring them that the information of individuals will remain confidential and that the general results of the research will be used in a research project. For the older adults who had visual problems and were unable to complete the questionnaire, the questions were read by the researcher, and their answers were recorded.

### Data and statistical analysis

Explanatory variables (i.e. socio-demographic, and injury profile) and loneliness score were tabulated using the statistical indicators of mean, standard deviation, and their frequencies. ANOVA followed by Scheffé’s post hoc test performed to determine whether loneliness scores differ between explanatory variables groups. The eta-square test was also used to measure the effect of variables on the loneliness index. Multivariate regression analysis performed possible relationships among variables that most contribute to the loneliness score of veterans and estimate the effect., Structural equation modeling (SEM) using the Maximum Likelihood Estimation (MLE) method was conducted to find the structural equations between variables and determine their degree of correlation (i.e. path coefficients). All statistical analyses were performed using the SPSS statistical program for Windows, version 16 (SPSS Inc., Chicago, IL, USA) and SPSS Amos (Analysis of Moment Structures, version 24). The significance threshold was P = 0.05 in all analyses.

### Ethical approval

The procedure followed in this research was approved by the independent Ethics Committee of Ahvaz Jundishapur University of Medical Sciences (Reference Code: IR.AJUMS.REC.1396.1115) before commencing the research.

## Results

According to the descriptive results of the present study, the largest sample population was in the age group of 50 to 60 years (55.6%), 62% of the participants were pensioners, and the distribution of education level across the population was the same (Mean = 5.87, SD = 4.96, n = 583). More than 68% of the veterans lived with their spouses and children. The average score of loneliness of the middle and older veterans was 51.8 with a standard deviation of 4.74, with the highest and lowest scores being 68 and 29, respectively (see Table 1).

**Table 1:** Sociodemographic Characteristics and loneliness of veterans in southern Iran (2021)

Variables	Domains	Frequency (%)	Loneliness Score		F test*	P-Value	ES**
			*Mean*	*SD*			
Age	50–60	324(55.6)	51.22	4.903	1.32	0.002	-0.26
	61–65	240(41.2)	50.37	5.114			
	66–70	19(3.3)	51.11	3.478			
Employment Status	Employee	128(22)	49.72	5.233	4.61	0.001	0.11
	Pensioner	364(62.4)	51.02	5.141			
	Self-employed	91(15.6)	51.86	3.325			
Education	Without high school diploma	200(34.3)	51.55	3.73	37.11	0.001	-0.31
	High school diploma	193(33.1)	48.32	3.97			
	University degree	190(32.6)	50.66	6.75			
Type of living	Living with spouse and children	400(68.6)	50.72	4.673	2.67	0.002	0.11
	Living with spouse alone	183(31.4)	51.17	5.545			
Residence province	Fars	211(36.2)	50.63	5.269	7.35	0.105	0.08
	Bushehr	20(3.4)	47.65	3.977			
	Hormozgan	101(17.3)	50.56	6.074			
	Kohgiloyeh and Boyerahmad	47(8.1)	51.79	3.917			
	Khuzestan	204(35)	51.36	4.147			
Total Score of Loneliness	All samples	583 (100)	50.87	4.96	247.47***	0.001	1.18

*One-Way ANOVA, ** ES = Effect Size using Eta-squared, *** One-sample t-test and using Cohen’s d for calculating effect size = 1.18, df = 582, 2-tailed Sig. ≤ 0.05

Veterans aged 61 to 65, had lower loneliness scores than their younger (< 60) and older (> 65) counterparts. Thus, who live with spouse alone, had higher total loneliness scores than those veterans accompanying their children. The study shows higher loneliness score among self-employed veterans than thus employees who work for an organization (effect size = 0.11, P = 0.001). Results indicate that compared to thus with no high school diploma, veterans with a medium or high education are less likely to feel lonely (effect size = 0.31, P-value = 0.001). Veterans of the Bushehr province experienced lower loneliness scores than other provinces however, the study could not find a significant difference in the average feeling of loneliness between the provinces (P-value = 0.105). The type of education with the highest impact factor of -0.31 had the reverse explanatory power on the level of loneliness.

As depicted in Table 2 and 34.8% of the middle and older veterans had less than 25% war-induced disability, 34% of them had 25–50% disability, and the rest of the veterans had more than 51% disability. More than half of veterans’ injuries or disabilities were amputated limbs (51.1%) while only a few participants were blind. The results showed that there was a significant difference between the severity of injury and the mean loneliness score in the middle and older veterans (P-value = 0.001). In other words, the severity of injuries had a bearing on the participants’ feelings of loneliness (effect size = -0.31). Also, there was a statistically significant difference between the mean of loneliness with different type of injury or disability (effect size = 0.31, P-value = 0.31). A significant difference between the severity of injury or disability and the feeling of loneliness observed mainly between veterans with less than 25% injury or disability and those with more than 51% injury or disability (p-value = 0.001). As far as the type of injury or disability was concerned, there was a significant difference in the mean score of the group of mentally disabled veterans and the mean score of those with vision disability (p-value = 0.04), affected by chemical warfare (p-value = 0.03) and with amputated limbs (p-value = 0.01). According to the Eta Square test, the severity and type of disability significantly associated with the level of social isolation (p = 0.001).

**Table 2:** Injury profile and loneliness score among the middle and older veterans, residents in the southwest of Iran

Variables	Domains	Frequency (%)	Loneliness Score		F test*	P-value	ES**
			*Mean*	*SD*			
Severity of injury or disability	Less than 25%	203(34.8)	51.85	4.079	8.36	0.001	-0.31
	Between 25–50%	198(34)	50.61	4.979			
	More than 51%	182(31.2)	50.05	5.642			
Type of injury or disability	Vision impairment	33(5.7)	48.76	3.674	5.63	0.001	0.31
	Affected by chemical warfare	105(18)	51.17	4.972			
	Amputated limb	298(51.1)	50.46	4.968			
	Spinal cord injury	41(7)	50.17	5.643			
	Neuropsychological trauma	106(18.2)	52.62	4.551			

*One-Way ANOVA, ** ES = Effect Size using Eta-squared

**Table 3:** Multivariate regression analysis for the association between loneliness and characteristics of veteran, southwest of Iran (2021)

Predictive Item	Source	Coefficient	SE	Beta	T	P-value
**Loneliness**	Age	-0.521	0.41	-0.26	-1.31	0.001
	Employment status	0.224	0.42	0.11	0.27	0.002
	Education	-1.734	0.37	-0.311	-5.13	0.001
	Type of living	1.172	0.52	0.110	3.31	0.002
	Severity of injury or disability	-0.567	0.37	-0.310	-1.05	0.001
	Type of injury or disability	0.558	0.28	0.313	2.17	0.002

*R = 0.482, R sqr = 0.232, adj R sqr = 0.230*

**Fig. 1:**
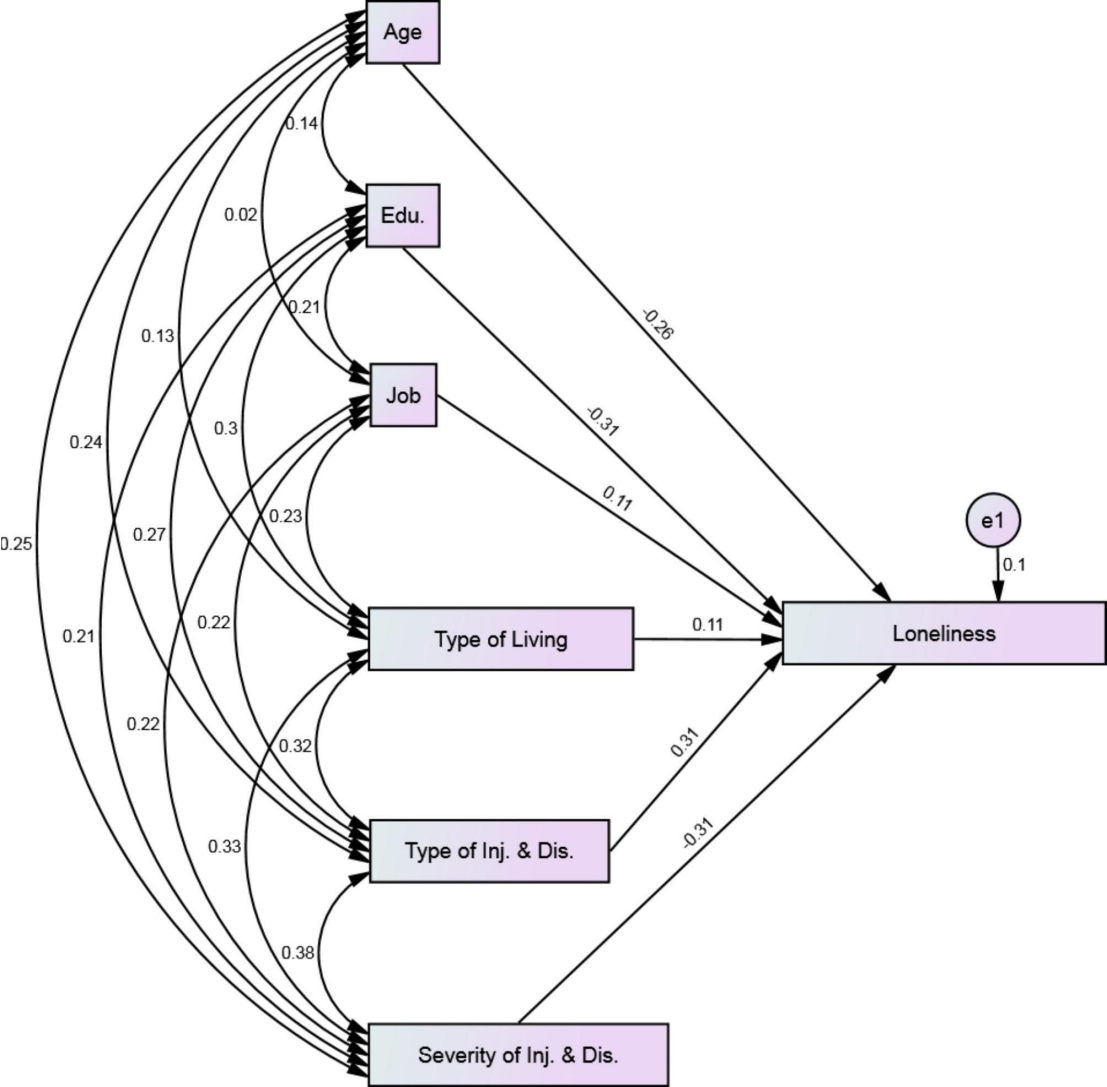
The SEM diagram of the extracted model

Education level (-0.31), severity of injury (-0.310), and type of injury (0.313) can predict the variance of loneliness in middle and older veterans. For example, the higher the level of education of veterans, the lower their score of feeling lonely (by 3.11 points). Also, living with their spouses alone increases their score of feeling lonely by 2.31 points (Table 3). According to Fig. 1, applying the SEM, the proposed model among 3 extracted models was approved with good fit indices. The type and severity of injury were the most explanatory factors of feeling lonely in veterans with a factor power of 0.31 and − 0.31, respectively (Χ2/df ≤ 3 = 1.874, RMSEA = 0.039, TLI = 0.89, CFI = 0.88).

## Discussion

The present study was performed on male veterans to investigate the extent and determinants of loneliness in southern Iran in 2021. The present study showed elevated levels of loneliness [17, 18]. These results are consistent with the results of Manaei et al. who studied psychiatric complications in blind veterans in Fars province [11]. However, no other regional or national studies reported the prevalence of loneliness.

The results of our study are also consistent with those of a study conducted on soldiers in the United States where a high prevalence of loneliness among these individuals was reported, and this figure was found to be far more than that of ordinary people. In this study, the feeling of loneliness was reported to be a strong predictor of the development of depression among the studied American soldiers [10] and the prevalence of psychiatric disorders [19]. Results of a 2020 study conducted on war veterans showed that a high measure of depression in all subjects [12]. Since veterans have numerous problems in their everyday life due to their physical and psychological injuries and the associated consequences, these problems, in most cases, elevated feelings of loneliness in them. In this regard, it can be argued that the reason for this increase can be attributed to the fact that the life of a disabled person brings about undue and undesirable mental and psychological consequences due to their inability to perform daily activities in society. Also, the highest measure is related to the age group of 50–60 years. In two similar studies conducted by Divanbeigi et al. [20] and Carr et al. [21], the mean age of veterans was reported to be 42.3 ± 5.7 years and 56.8 ± 4.28, respectively. This difference between age groups can be attributed to the fact that younger age groups have been more exposed to this complication due to their active participation and physical abilities in the wartime.

In terms of the employment status the present study, more than half of veterans were pensioners and retirees, and here was a statistically significant difference between the employment status and the feeling of loneliness. The reason for this difference could be that various psychological factors such as boredom, stress, depression, and lack of social interaction in the workplace, the greater severity of physical problems, disability, and the severity of the injury, all of which increase the rate of loneliness among veterans. This finding is consistent with that of Werum et al., where more than 70% of the participants were not employed. Employment and being active are among the factors that can contribute to restoring the abilities of veterans and maintaining their morale [22]. Tao et al. reported that there is a positive and significant relationship between job satisfaction and mental health among chemical warfare victims [23]. In fact, job satisfaction is a complex concept that is associated with various psychological and social factors; in other words, different factors play a major role in creating job satisfaction or dissatisfaction. For instance, a previous study has found that stress is associated with a negative effect on mental health, hence reducing job satisfaction [24].

Concerning the education level, the highest percentage of the samples did not have education beyond reading and writing. There was also a statistically significant difference between the levels of education and loneliness among them. The results of a study by Blau et al. showed mental health of veterans with university degrees being significantly different from that of other veterans [25]. In this respect, a previous study dealing with veterans’ quality of life reported that higher level of education affects their quality of life, which is in turn affected by their mental health [26].

Another finding of the present study was a significant relationship between the severity of injury or disability and the mean score of loneliness. This means that 34.8% of the middle and older veterans with less than 25% injury or disability had the highest frequency whereas 31.2% of them with more than 50% injury or disability had the lowest frequency. The reason for this difference could be that physical activity and mental health problems, including depression and loneliness, increase in veterans who have a more severe injury or disability (i.e., these mental health problems increase as their injury or disability rate increases). In the study of Bigelow et al., it was found that the highest frequency of psychological trauma was in veterans with high disabilities. In other words, the higher the rate of disability, the greater the psychological trauma among these people [27]. Another study found a statistically significant relationship between the rate of disability with depression, anxiety, and insomnia [28].

In the present study, the majority of the middle and older veterans lived with their spouses and children and 31.4% with their spouses alone, indicating no statistically significant difference between the feeling of loneliness and the type of living. Schwartz et al., however, reported that the average general health scores were higher in people who lived with their spouse and children, which indicates the positive effect of living with family compared to living alone [29]. As far as the type of injury or disability was concerned in the present study, the highest frequency was related to amputee veterans while the lowest rate was related to those with vision impairments. There was a statistically significant difference between the mean of loneliness and different groups of veterans. The results of Delpisheh study showed that in terms of injured limbs, 29% of the veterans in their study had sustained upper extremity injuries and 22% of them had lower extremity injuries, and in terms of the type of injury or disability, 35% of the veterans were chemical warfare victims, 14% had neuropsychological trauma, and the most of them had spinal cord injury, the most common determinant of which was shrapnel by 51.4% [30]. Veterans with physical disabilities followed by those suffering from neuropsychological problems had the highest frequency [31]. Therefore, considering the mental health status of middle and older veterans, the following strategies can promote public mental health in general and the mental health of the veterans: increasing social support, offering psychiatric counseling sessions for veterans, improving their income and the facilities available for them, allocating allowances based on the difficulty of their work in different workplace settings. Despite the mentioned limitations and relying on the results of the present study, the following suggestions can be made. First, to promote the mental health of veterans, they need to be encouraged to participate in group and collective activities. In this way, they can help improve their mental health and reduce their stress. Second, to help the veterans elevate their mental health status, training courses can be used to focus on increasing motivation, self-development, and resilience. Finally, serious planning should be done to promote sports activities among veterans. Definitions of severity of injury among veteran vary across countries which may limit the generalizability of the results to other dominions. Even though access to the closed and conservative society of Iranian veterans in a wide geographic population in Iran was one of the strengths of this study, social context and living situation in north and eastern regions of country differ. Further surveys and outreach may consider collecting more in-depth information to be conducted in other parts of Iran.

## Data Availability

The datasets used during the current study are available upon reasonable request.
